# CCL1 blockade alleviates human mesenchymal stem cell (hMSC)-induced pulmonary fibrosis in a murine sclerodermatous graft-versus-host disease (Scl-GVHD) model

**DOI:** 10.1186/s13287-020-01768-7

**Published:** 2020-06-26

**Authors:** Ji-Young Lim, Da-Bin Ryu, Tae Woo Kim, Sung-Eun Lee, Gyeongsin Park, Hyoung Kyu Yoon, Chang-Ki Min

**Affiliations:** 1 Hematology, Department of Internal Medicine, Seoul St. Mary’s Hospital, The Catholic University of Korea, 222 Banpodae-ro, Seocho-gu, Seoul, 06591 South Korea; 2 Department of Pathology, Seoul St. Mary’s Hospital, The Catholic University of Korea, Seoul, South Korea; 3 Pulmonology, Department of Internal Medicine, Seoul St. Mary’s Hospital, The Catholic University of Korea, Seoul, South Korea

**Keywords:** Mesenchymal stem cells, Chronic graft-versus-host disease, Sclerodermatous graft-versus-host disease, Allogeneic hematopoietic stem cell transplantation

## Abstract

**Background:**

Human chronic graft-versus-host disease (CGVHD) shares clinical characteristics with a murine sclerodermatous GVHD (Scl-GVHD, B10.D2 → BALB/c) model that is characterized by skin and lung fibrosis. In this study, bone marrow- or adipose tissue-derived human mesenchymal stem cells (hMSCs) were injected into the Scl-GVHD mice to address their therapeutic effect on CGVHD.

**Methods:**

Lethally irradiated BALB/c mice were transplanted with B10.D2 T cell-depleted bone marrow with or without spleen cells to generate Scl-GVHD. hMSCs were intravenously treated on days 3, 5, and 7 post-transplantation, and the control antibody or CCL1 blocking antibody was subcutaneously injected according to the same schedule as the hMSCs. Fourteen days after transplantation, the recipient mice were sacrificed, and their skin and lungs were analyzed.

**Results:**

After the early injection of hMSCs after transplantation, the clinical and pathological severity of Scl-GVHD in the skin was significantly attenuated, whereas the pathological score was exacerbated in the lungs. hMSCs had migrated into the lungs, but not into the skin. CD11b monocyte/macrophages and CD4 T cells were markedly decreased in skin tissues, whereas there was an early recruitment of CD11b cells, and subsequently increased infiltration of CD4 T cells, in the lungs. Importantly, hMSCs persistently upregulated the expression of CCL1 in the lungs, but not in the skin. Concurrent treatment of hMSCs with a CCL1-blocking antibody alleviated the severity of the lung histopathology score and fibrosis with the preservation of the cutaneous protective effect against CGVHD. Infiltration of CD3 T cells and CD68 macrophages and upregulation of chemokines were also decreased in lung tissues, along with the recruitment of eosinophils and tissue IgE expression. In the skin, chemokine expression was further reduced after CCL1 blockade.

**Conclusions:**

These data demonstrate that despite a protective effect against Scl-GVHD in the skin, administration of hMSCs exacerbated lung fibrosis associated with eosinophilia and airway inflammation through persistent CCL1 upregulation. CCL1 blockade offers a potential treatment of pulmonary complications induced after treatment with hMSCs.

## Background

In connection with the rising use of allogeneic hematopoietic stem cell transplantation (allo-HSCT) in elderly patients, the prevalent use of mobilized blood cells instead of marrow as the stem cell source, and decreased mortality rate during the early stages of allo-HSCT [1, 2], chronic graft-versus-host disease (CGVHD) has risen over the past 20 years both in prevalence and severity, and yet, currently accessible therapies with long-term immunosuppressive drugs have shown only limited efficacy. The most common pathological changes in human CGVHD are sclerodermatous GVHD (Scl-GVHD), which affects nearly all organs and tissues and is exhibited in a considerable increase in collagen deposition, mainly presenting lichen planus, sclerotic lesions, pigment disorders, and leopard skin differentiation [3–5]. Very few experimental models exist to examine CGVHD [6]. Among them, the B10.D2 (H-2^d^) → BALB/c (H-2^d^), major histocompatibility complex (MHC)-matched and minor histocompatibility antigen-mismatched model, is able to replicate human Scl-GVHD and systemic sclerosis, primarily through the thickening of the skin and pulmonary fibrosis resulting after increased excessive collagen due to extracellular matrix deposition [7–9].

Mesenchymal stem cells (MSCs) and stromal cells that typically reside within the adult bone marrow can differentiate into many adult cell types, such as osteocytes, chondrocytes, and adipocytes [10]. It was previously observed in the murine Scl-GVHD model that allogeneic murine MSCs (mMSCs), by selectively blocking immune cell migration and downregulating chemokines and chemokine receptors, mitigate the severity of cutaneous Scl-GVHD [11]. hMSCs possess immunomodulatory properties and an ability to produce soluble proteins that critically support hematopoietic stem cell homeostasis and engraftment [12–14]. Given the numerous differences in immunomodulatory effects between the two MSCs, our study chose to focus on hMSCs in order to improve clinical applicability. Although hMSCs have been clinically used for steroid-refractory acute GVHD [15] due to their immunosuppressive properties, ease in expansion, and safe infusion profile [16], the protection against Scl-GVHD, bio-distribution, and mechanisms underlying in vivo hMSC effects remain largely undefined. Murine xenogeneic transplant models might prove to be suitable for defining in vivo hMSC-mediated immunosuppression, owing to hMSCs’ low immunogenicity, lack MHC class II and co-stimulatory molecule expression, and failure to activate T cells in vitro [17]. With that considered, we tested the hypothesis that hMSCs would mitigate the severity of Scl-GVHD in mice after allo-HSCT. Our findings did reveal hMSCs to effectively diminish Scl-GVHD in skin tissue; dissimilarly, hMSCs resulted in a localization of Scl-GVHD in injured lungs, which worsened pulmonary fibrosis. This fibrotic response in the lungs was caused by hMSC elicitation of immune cell migration due to the upregulation of CCL1. In summary, we found that hMSCs exerted a paradoxically different effect on skin and lung tissues in an established Scl-GVHD model, which may offer up a treatment strategy to lessen the severity of cutaneous Scl-GVHD while lowering pulmonary complications.

## Methods

### Experimental allo-HSCT and MSCs

Female B10.D2 (H-2^d^) and BALB/c (H-2^d^) mice (8 to 12 weeks old) were purchased from Shizuoka Institute for Laboratory Animals (Japan SLC, Shizuoka, Japan). Briefly, recipient (BALB/c) mice were lethally irradiated with 650 cGy using a Gammacel^137^Cs source. Approximately 6 h later, they were injected i.v. via the tail vein with donor (B10.D2) T cell-depleted bone marrow (5 × 10^6^ cells/mouse) and spleen cells (3 × 10^6^ cells/mouse) (referred to as Scl-GVHD mice). A control group of BALB/c recipient mice received either B10.D2 donor BM without T cells (non-GVHD controls) or BALB/c BM with T cells (syngeneic controls). The primary human BM and AD MSCs were obtained from a stem cell bank. BM MSCs (Scl-GVHD + hBM MSCs) or AD MSCs (Scl-GVHD + hAD MSCs) were administered after allo-HSCT at a dose of 3 × 10^5^ cells/mouse.

### GVHD skin score and histopathological analysis

The clinical skin GVHD score was modified as previously described [7]. The minimum score was 0, and the maximum score was 8. Formalin-fixed, paraffin-embedded tissue sections were subjected to hematoxylin-eosin (H&E) staining for microscopic examination and Masson’s trichrome staining for fibrosis. Slides were scored by a pathologist (blinded to experimental groups). Dermal thickening from the bottom of the epidermis to the fat was evaluated for each animal as previously described [18]. The Masson’s trichrome-stained section was standardized for the photographic area (1200 × 1200 pixels) allowing a direct comparison between images. The collagen area was traced and measured using the ImageJ software (http://rsb.info.nih.gov), and the percentage of collagen per standardized field of view (× 100 magnification) was calculated.

### Protein extracts and measurement of soluble collagen and IgE

Tissue samples were immediately frozen in liquid nitrogen, disrupted using a Polytron homogenizer (pellet pestles cordless motor, Sigma), and centrifuged at 3000 rpm for 20 min. Proteins were purified from the supernatant, and the concentration was assessed using the Bradford method (Bio-Rad, Hercules, CA). Total soluble collagen was quantified using the Sircol Soluble Collagen Assay (Bio-color, Belfast, Ireland) as previously described [19]. Concentrations of IgE were determined with a Mouse IgE ELISA MAX Standard kit (Biolegend, 432401).

### Immunohistochemical staining

Tissue sections (4 μm) were mounted on super frost glass sliders and deparaffinized in xylene and a graded series of ethanol, followed by antigen retrieval. Endogenous peroxidase activity was blocked with 3% hydrogen peroxide. Non-specific binding sites were saturated by exposure to 10% normal goat serum diluted in PBS for 60 min. Slides were then incubated overnight at 4 °C with primary antibodies against mouse MMP1 (1:250 dilution, Abcam), PTEN (1:100 dilution, Abcam), pSmad3 (1:200 dilution, Invitrogen), CD3 (1:400 dilution, Santa Cruz), CD68 (1:250 dilution, Abcam), or EPX (1:200 dilution, Abcam) then washed with PBS for 10 min. Biotinylated goat anti-rabbit IgG and rabbit anti-goat IgG (Vector Laboratories, Burlingame, CA) secondary antibodies were applied to tissue sections, and the slides were incubated at room temperature for 30 min. After the sections were washed and incubated for 30 min with peroxidase-conjugated streptavidin (Dako, Glostrup, Denmark) at room temperature, 3,3′-diaminobenzidine was added to visualize antigens. Sections were counterstained with Mayer’s hematoxylin, dehydrated, cleared, and mounted. Negative control tissue samples were prepared in the same manner as described above, except that the primary antibody was omitted or replaced with an isotype control antibody (R&D Systems, Minneapolis, MN).

IHC stains were evaluated for the presence of positively staining cells in the dermis as previously described [20]. In brief, the following semiquantitative scale, based on the percentage of positive cells, was used: 0 (no staining), 1 (< 25% staining), 2 (25–50% staining), 3 (50–75% staining), or 4 (75–100% staining). Stained cells were counted under a high-power microscopic field (× 400 original magnification) on a light microscope.

### Fluorescent detection for in vivo tracking of MSCs

Primary hMSCs were labeled with PKH-26 according to the manufacturer’s instructions (Sigma-Aldrich, St. Louis, Mo, USA) and injected into the recipient mice. To evaluate the MSC recruitment, tissues were immediately embedded in the OCT (CellPath) embedding matrix, placed on dry ice, and stored at − 80 °C. Tissues were then sectioned on a cryostat (4 μm), fixed with acetone, and, after washing, stained with 4,6-diamidino-2-phenylindole (DAPI; Sigma-Aldrich). Slides were examined using a confocal microscope (LSM700; Carl Zeiss).

### Quantitative real-time PCR analysis

Total RNA was isolated from skin and lung homogenates with TRIzol (Invitrogen, Carlsbad, USA) in accordance with the manufacturer’s instructions. One microgram of total RNA was reverse transcribed into cDNA. Quantitative assessment of target mRNA levels was performed by quantitative RT-PCR using a CFX96TM real-time PCR detection system (Bio-Rad, Hercules, CA, USA). Results were normalized to β-actin expression and expressed as fold change compared with non-GVHD control mice, where specified, using the 2^−ΔΔCt^ method. The sequences of forward and reverse primers are shown in Table 1.

**Table 1:** The sequences of forward and reverse primers

Gene	Forward sequence (5′-3′)	Reverse sequence (5′-3′)
Col1α1	CAGTCGATTCACCTACAGCACG	GGGATGGAGGGAGTTTACACG
Col1α2	GGAGGGAACGGTCCACGAT	GAGTCCGCGTATCCACAA
Col3α1	GTTCTAGAGGATGGCTGTACTAAACACA	TTGCCTTGCGTGTTTGATATTC
CCL1	CGTGTGGATACAGGATGTTGACAG	AGGAGGAGCCCATCTTTCTGTAAC
CCL2	CTCACCTGCTGCTACTCATTC	GCTTGAGGTGGTTGTGGAAAA
CCL3	ACTGCCTGCTGCTTCTCCTACA	AGGAAAATGACACCTGGCTGG
CCL5	TGCCCACGTCAAGGAGTATTTC	AACCCACTTCTTCTCTGGGTTG
CCL8	TCTACGCAGTGCTTCTTTGCC	AAGGGGGATCTTCAGCTTTAGTA
CCL17	ACCGCTCATCTGTGCAGACC	CGCCTGTAGTGCATAAGAGTCC
CCL22	AAGACAGTATCTGCTGCCAGG	GATCGGCACAGATATCTCGG
CCR4	GCTTGGTCACGTGGTCAGTG	GTGGTTGCGCTCCGTGTAG
CCR8	CAGATAATTGGTCTTCCTGCCTC	TGAGGAGGAACTCTGCGTCACA
β-Actin	AGCTGCGTTTTACACCCTTT	AAGCCATGCCAATGTTGTCT

### Bronchoalveolar lavage

Bronchoalveolar lavage (BAL) was performed in situ four times with Hanks’ Balanced Salt Solution (35 ml/kg; pH 7.2–7.4), and the recovered BAL fluid (BALF) was immediately cooled in ice. BALF returns were centrifuged (1500 rpm, 5 min at 4 °C), and the pooled cell pellets from all BALF were combined and resuspended in 1 ml of Hanks’ Balanced Salt Solution, and the number of BAL cells was counted with a hemacytometer. Aliquots (100 μl) of cell resuspension were cytocentrifuged, and the cytospin slides were stained with Diff-Quick for differential cell counting (markers of cellular inflammation and epithelial injury). Percentage and absolute numbers of each cell type were calculated in triplicate.

### Cell isolation and flow cytometric analysis

Mononuclear cells were isolated from the skin as previously described [18]. Briefly, minced skin was digested for 30 min in a complete medium supplemented with Liberase and DNase (both purchased from Roche), and leukocytes were isolated by density gradient centrifugation on AccuPrep medium (Accurate Chemical, Oslo, Norway). To determine the surface phenotype, single-cell suspensions were stained with PE-conjugated anti-CD11b and APC-Cy7-conjugated anti-CD4 at 4 °C for 30 min. Samples were analyzed using an LSRII (BD Pharmingen, San Diego, CA).

### Statistical analysis

All values are expressed as mean ± standard error of the mean (SEM). Statistical comparisons between the groups were performed using a parametric independent sample *t* test if there were > 5 animals per group or using the Mann-Whitney test if there were < 5 animals per group.

## Results

### Characteristics of hMSCs

hMSCs are characterized by the expression of several surface markers and display multipotent differentiation along mesenchymal lineages. We determined the phenotype of hMSCs by flow cytometry. Cells were positive for MSC markers (CD44, CD73, and CD166). In addition, the cells were negative for hematopoietic markers (CD34, CD45, CD14, CD11b) and HLA-DR (Supplementary Figure 1a). To determine whether hMSCs could differentiate into multiple mesenchymal cell lineages, hMSCs were cultured in osteogenic, adipogenic, and chondrogenic medium. The qualitative confirmation of differentiation was made by alizarin red staining for osteogenic differentiation, oil red o staining for adipogenic differentiation, and alcian blue staining for chondrogenic differentiation (Supplementary Figure 1b).

### Early injection of hMSCs attenuated the severity of murine Scl-GVHD in the skin but exacerbated pulmonary inflammation and fibrosis

**Fig. 1:**
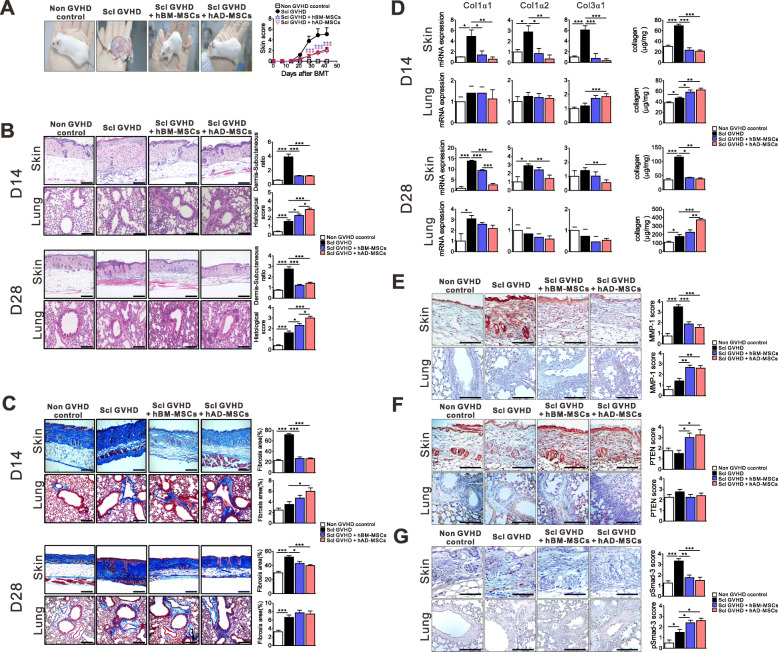
Human mesenchymal stem cells (hMSCs) attenuated the severity of skin sclerodermatous graft-versus-host disease (Scl-GVHD) but exacerbated pulmonary inflammation and fibrosis. **a** BALB/c mice transplanted with T cell-depleted bone marrow (TCD-BM) and spleen cells from B10.D2 mice had chronic dermatitis and an increased average skin score (Scl-GVHD). However, mice receiving human bone marrow (Scl-GVHD + hBM MSCs)- or adipose tissue (Scl-GVHD + hAD MSCs)-derived hMSCs had markedly decreased chronic dermatitis and skin scores. BALB/c mice transplanted with cells from B10.D2 TCD-BM (non-GVHD control) did not show dermatitis or hair loss. **b** Paraffin-embedded tissue sections were stained with H&E (original magnification × 100) for microscopic examination. **c** Masson’s trichrome staining (original magnification × 100) for assessing fibrosis. **d** mRNA expression of collagen 1 α1, 1 α2, 3 α1, and soluble collagen were measured on day 14 (upper panel) and day 28 (lower panel) after transplantation. **e**–**g** Immunohistochemical staining of MMP1 (**e**), PTEN (**f**), and pSmad-3 (**g**) was quantified on day 14 after transplantation. Original magnification, × 200. Each value indicates the mean ± SEM of 4–9 mice per group. *P < 0.05; **P < 0.01, ***P < 0.001

Bone marrow (BM)- or adipose tissue (AD)-derived hMSCs were intravenously administered to allogeneic recipients on days 3, 5, and 7 after experimental allo-HSCT. The clinical severity of Scl-GVHD in the skin was significantly attenuated in hBM or hAD MSC-treated recipients relative to Scl-GVHD controls (Fig. 1a). Histologic examination revealed thickening of the epithelial layer, loss of hair follicles and subdermal fat, and ulcers in the epithelial and dermal layers in skin lesions of Scl-GVHD controls. The pathological severity of Scl-GVHD in the skin but not the lungs was significantly attenuated in either hBM or hAD MSC-treated recipients relative to Scl-GVHD controls at days 14 and 28 (Fig. 1b).

Moreover, in both BM and AD hMSC-treated groups, fibrosis areas and collagen amounts in the skin but not in the lungs were significantly lower than those in the allogeneic control group (Fig. 1c). In parallel, the mRNA expression levels of collagen type 1 α1 (*COL1A1*), 1 α2 (*COL1A2*), and 3 α1 (*COL3A1*) were significantly reduced in skin tissue after both hBM and hAD MSC treatments, compared to those in allogeneic Scl-GVHD controls. In contrast, those parameters were largely increased in lung tissue after both hBM and hAD MSC treatments (Fig. 1d). Type I collagen, the most abundant extracellular matrix (ECM) protein, is deposited excessively in the dermis of scleroderma. Matrix metalloproteinases (MMPs) play a major role in ECM degradation [21]. In injured human skin, MMP-1 is induced as wound-edge keratinocytes bind type I collagen in the dermis, and the ability of this proteinase to cleave to collagen is key to facilitating keratinocyte movement [22]. MMP-1 expression in the skin was reduced in hMSC-treated allogeneic recipients compared with Scl-GVHD controls. However, the expression of MMP-1 was significantly increased in lung tissue after hMSC treatment (Fig. 1e). TGF-β plays a central role in pathological tissue fibrosis. TGF-β triggers the activation of numerous signaling pathways, which contribute to progressive fibrosis, including the Smad pathway [23]. TGF-β induces the profibrotic activity via suppression of the PTEN expression [24]. hBM and hAD MSC treatment restored PTEN expression and reduced the level of Smad-3 phosphorylation in the skin (Fig. 1f, g, respectively). In the lungs, however, the levels of PTEN did not differ between hMSC-treated allogeneic recipients and Scl-GVHD controls, and Smad-2/3 phosphorylation was increased in BM and AD hMSC-treated allogeneic recipients (Fig. 1f, g, respectively).

### In vivo detection of injected hMSCs in each organ and infiltration of immune effector cells into the skin and lungs

**Fig. 2:**
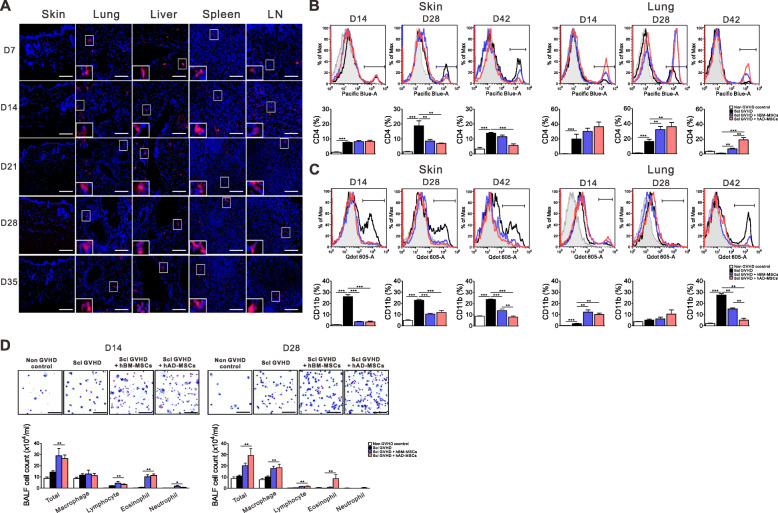
Migration of hMSCs into each organ and analysis of immune cell infiltration into skin and lung tissues. **a** PKH-26-labeled hAD-MSCs were injected into the tail vein on 3, 5, and 7 days after allo-HSCT. The skin, lung, liver, spleen, and lymph node (LN) sections were analyzed by confocal microscopy for the detection of PKH-26-positive cells (shown in red). Flow cytometric analyses were performed using skin and bronchoalveolar lavage fluid (BALF). **b**, **c** Frequency of CD4 T cells (**b**) and CD11b (**c**) cells 14, 28, and 42 days after transplantation. **d** Total BALF cell count 14 and 28 days after transplantation. Each value indicates the mean ± SEM of 4–9 mice per group. *P < 0.05; **P < 0.01, ***P < 0.001

To examine the life span of hMSCs in GVHD target organs and secondary lymphoid organs, allogeneic recipients receiving AD hMSCs were sacrificed at 7, 14, 21, 28, and 35 days after allo-HSCT. hMSCs were frequently observed in the lungs, liver, spleen, and peripheral lymph nodes (LN), but not the skin (Fig. 2a). It has been noted that the initial target organ inflammation is caused primarily by CD11b monocytes and T cells (Hamilton al., 1087). We performed flow cytometric analysis of skin cell suspensions and BAL fluid using CD4 and CD11b surface markers. The frequency of CD4 T (Fig. 2b) and CD11b cells (Fig. 2c) was markedly increased in skin cells and BAL fluid following allo-HSCT. After BM and AD hMSC treatment, the frequencies of CD4 T and CD11b cells in the skin were markedly decreased compared to those in Scl-GVHD controls. On the other hand, there was an early accumulation of CD11b cells followed by the increase in infiltrating CD4 T cells thereafter. Leukocyte proportion in BAL fluid was enumerated by cytospin, and eosinophils were significantly increased after treatment with hMSCs in Scl-GVHD mice (Fig. 2d).

### Injection of hMSCs elicited differential expression of chemokines between the skin and lungs

**Fig. 3:**
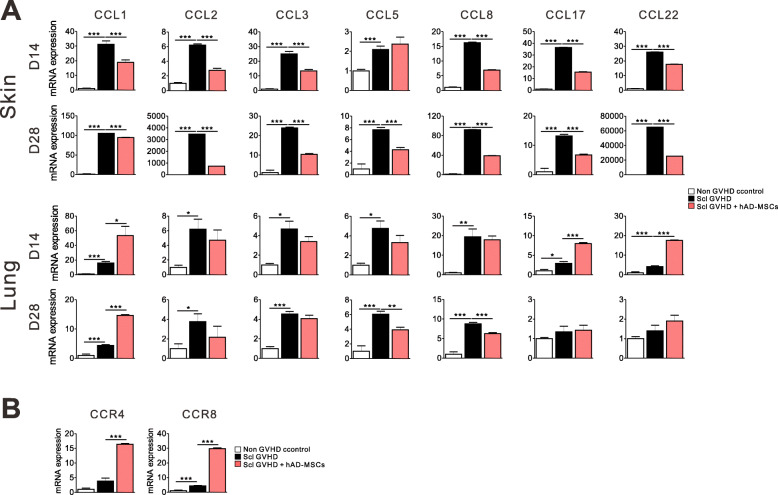
The different expression of chemokines after hMSC treatment in the skin and lungs. **a** Level of mRNA for CCL1, CCL2, CCL3, CCL5 CCL8, CCL17, and CCL22 in the skin and lungs 14 and 28 days after transplantation. **b** mRNA expression of CCR4, CCL17, and CCL22 receptor, and CCR8 and CCL1 receptor in the lungs 14 days after transplantation. Each value indicates the mean ± SEM of 4–9 mice per group. *P < 0.05; **P < 0.01, ***P < 0.001

Chemokines play important roles in recruiting cells of the innate and adaptive immune system to sites of inflammation. Several studies have now described the increased expression of chemokine receptors in GVHD [25]. The profile of chemokines and their receptor expression differs according to the target organs [26]. In the skin and lungs, mRNA for chemokines CCL1, CCL2, CCL3, CCL5, CCL8, CCL17, and CCL22 was upregulated in Scl-GVHD controls. As shown in Fig. 3a, chemokine mRNA expression was significantly reduced by hMSCs in the skin, however hMSCs upregulated the expression of Th2 chemokines, such as CCL1, CCL17, and CCL22 in the lungs. Among them, enhanced CCL1 mRNA expression in the lungs was maintained up to 28 days after transplantation. CCL1 is the only known ligand for CCR8, whereas CCL22 and CCL17 both interact with CCR4 [27]. In the lungs, mRNA expression of CCR4 (CCL17 and CCL22 receptor) and CCR8 (CCL1 receptor) was also significantly increased after hMSC treatment (Fig. 3b).

### Concurrent treatment with hMSCs and CCL1-blocking antibody alleviated the severity of lung histopathology score and fibrosis with preservation of the CGVHD protective effect in the skin

**Fig. 4:**
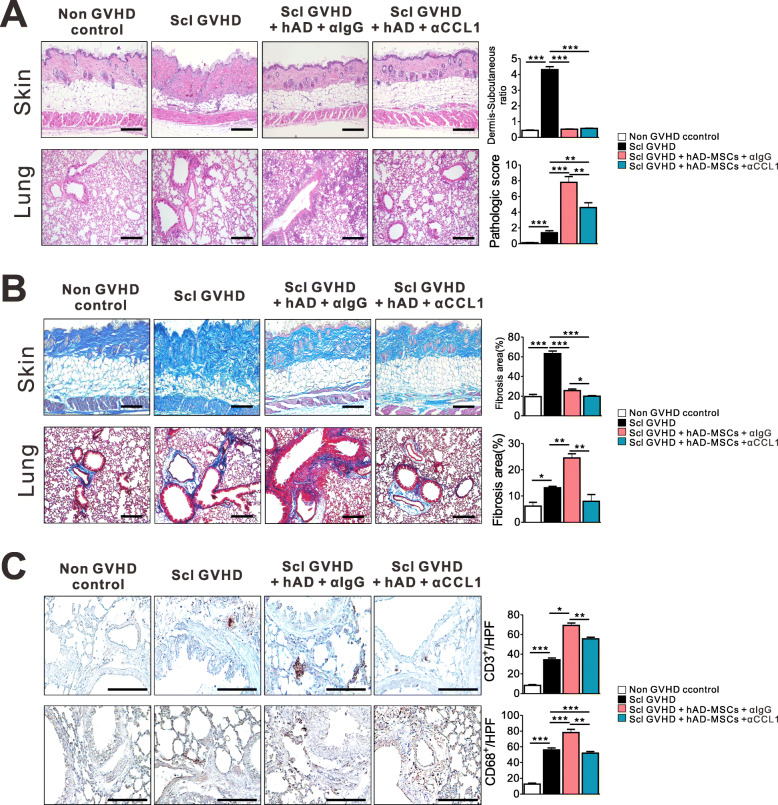
CCL1 blockade attenuated the severity of lung histopathology score and fibrosis while maintaining protective effects against CGVHD in the skin. **a**, **b** Representative photomicrographs of histopathological changes from non-GVHD controls, Scl-GVHD, Scl-GVHD + hAD MSCs + αIgG, and Scl-GVHD + hAD MSCs + αCCL1 groups on day 20. The sections were stained with H&E (**a**) or Masson’s trichrome (**b**). **c** CD3^+^ T cell and CD68^+^ macrophage infiltration of lung tissues was examined in each group. Each value indicates the mean ± SEM of 4–9 mice per group. *P < 0.05; **P < 0.01, ***P < 0.001

To further investigate whether CCL1 plays a role in the pathogenesis of pulmonary inflammation induced by hMSCs, we evaluated whether neutralization of CCL1 attenuated pulmonary inflammation without affecting the skin. Treatment with CCL1-neutralizing Abs reduced the severity of the lung pathological score while maintaining the protective effect against Scl-GVHD in the skin (Fig. 4a). Moreover, treatment with CCL1-blocking Ab also reduced pulmonary fibrosis induced by hMSCs (Fig. 4b) as compared with control Ab-treated mice. Next, we investigated whether CCL1 blockade could mitigate the infiltration of effector cells in target organs. CCL1 blockade reduced the infiltration of CD3 T cells and CD68 macrophage in lung tissues (Fig. 4c).

### CCL1 blockade reduced chemokine expression linked to airway infiltration of Th2-based CD4 T cells as well as eosinophil infiltration

**Fig. 5:**
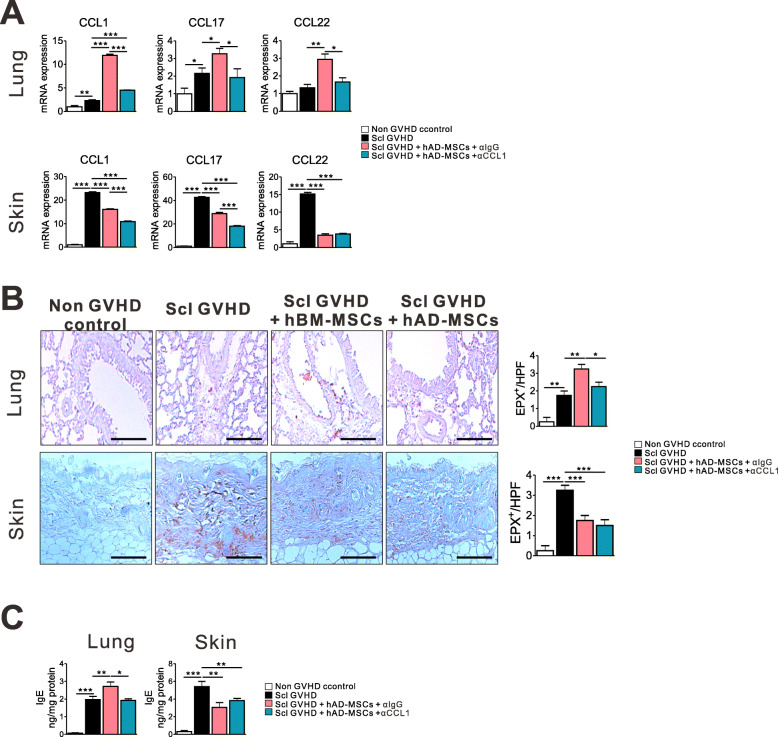
CCL1 blockade reduced Th2-related chemokine expression as well as eosinophil infiltration. **a** Level of mRNA for CCL1, CCL17, and CCL22 in the lungs and skin on day 20. **b** Eosinophil peroxidase (EPX) immunohistochemical staining was quantified on day 20. Original magnification, × 200. **c** IgE production in the lungs and skin on day 20. Each value indicates the mean ± SEM of 4–9 mice per group. *P < 0.05; **P < 0.01, ***P < 0.001

Th2 cells have been identified as the cells involved in controlling immunoglobulin E (IgE) production and influencing the functions of eosinophils [28]. We investigated whether CCL1 blockade could reduce Th2 chemokine expression and eosinophil infiltration induced by hMSCs. Upregulated chemokines, such as CCL1, CCL17, and CCL22, were also decreased after anti-CCL1 treatment in lung tissues. Moreover, the expression of CCL1 and CCL17 in the skin was further reduced after concomitant administration of hMSCs and anti-CCL1 compared to hMSCs alone (Fig. 5a). Next, we analyzed eosinophil infiltration after CCL1 blockade using eosinophil peroxidase (EPX) staining in target tissues. EPX is a major constituent of the large cytoplasmic granules of eosinophilic leukocytes. Treatment with CCL1-blocking Ab reduced eosinophil infiltration in the lungs compared to control Ab-treated mice (Fig. 5b). In parallel, CCL1 blockade also reduced hMSC-induced IgE production in lung tissues (Fig. 5c).

## Discussion

Ex vivo-expanded MSCs derived from various tissue sources can be used in both autologous and allogeneic settings for the evaluation of therapeutic efficacy. Multiple clinical studies have confirmed the safety of both allogeneic and autologous MSCs for the treatment of various human diseases [16]. The immune evasive and immunomodulatory properties of MSCS allow them to avoid immunologic rejection in an unrelated recipient and render them suitable for allogeneic or even xenogeneic cell therapy [29, 30]. Therefore, comprehensive preclinical safety and toxicity studies are needed before administering these cells into humans. In particular, the use of hMSCs to prevent or treat CGVHD with sclerodermatous changes has been limited by an incomplete understanding of the mechanism of action of these cells and unverified risks. In this study, we showed that administration of AD- or BM-derived hMSCs early after experimental allo-HSCT clearly reduced the severity of cutaneous GVHD of allogeneic recipients, as determined by both skin manifestation and histopathology. Yet, hMSCs also markedly exacerbated the inflammation and fibrosis in the lungs of Scl-GVHD mice. hMSCs specifically decreased the infiltration of both CD4 T and CD11b effector cells into the skin, but led to an increase in CD11b cells, particularly eosinophils, infiltrating into the lungs, followed by CD4 T cells. Expression of chemokines and chemokine receptors was different between the two organs, and we reasoned that continuous upregulation of CCL1 in the lungs induced by hMSCs could be related to pulmonary fibrosis with eosinophilia and airway inflammation. Concurrent treatment of hMSCs with a CCL1-blocking antibody reduced the effector cell infiltration and improved lung injury without attenuation of the protective effect against CGVHD on the skin. These results suggest that CCL1 blockade may be a potential treatment of pulmonary complications induced after hMSC treatment in clinics.

It is unexpected that hMSC treatment exacerbated the pathologic severity in the lungs because the lungs are the first homing site for MSCs after intravenous injection [31, 32]. It has been reported that intravenously infused MSCs home to the lungs in C57BL/6 recipient mice and induce an inflammatory response [33]. Since the Scl-GVHD mice are already in an inflammatory state, it is possible that the inflammatory response of the lungs has been accelerated after the injection of hMSCs.

In this murine Scl-GVHD model [34], the infiltration of mononuclear cells (predominantly CD4 T cells and CD11b monocyte/macrophages) into target organs starts 14 days after allo-HSCT [8, 11, 35]. After the injection of hMSCs, the infiltration of mononuclear cells was profoundly reduced into the skin, whereas in the lungs, an increase in infiltrating CD4 T cells followed the early accumulation of CD11b cells. The entry of leukocytes into lymphoid and non-lymphoid tissues is controlled by sequential engagement of inflammatory cytokines and/or chemokines [36, 37]. It has been previously shown that mMSCs attenuate cutaneous Scl-GVHD by selectively blocking immune cell migration and downregulating chemokines and chemokine receptors [11]. We wondered whether chemokine expression pattern was a factor determining the preferential organ involvement after early injection of hMSCs, which led us to analyze chemokine receptors involved in lung diseases [38]. After hMSC infusion, elevated expression of skin profibrotic chemokines, such as CCL1, CCL2, CCL3, CCL8, CCL17, and CCL22, was downregulated, consequently resulting in decreased infiltration of immune cells into skin tissues. In contrast, hMSCs enhanced the expression of CCL1 with CCR8, CCL17, and CCL22 with CCR4 in the lungs, contributing to the early recruitment of CD11b cells, particularly eosinophils, to the lungs. CCL1 binds exclusively to CCR8, whereas CCL22 and CCL17 both interact with CCR4 [27]. We found that hMSCs early after transplantation allowed eosinophils to be recruited into the lungs, which played a major role in pulmonary inflammation and fibrosis. Neutralizing either CCL22 or CCL17 has been shown to abrogate lung eosinophilia and airway hyperresponsiveness [39, 40]. Direct instillation of CCL1 into the lungs of allergen-sensitized challenged mice induces the recruitment of eosinophils, and CCL1 blockade induces a modest reduction in eosinophil recruitment [41]. In our study, CCL1 blockade had a beneficial effect on hMSC-induced pulmonary complications by suppressing the infiltration of CD3 and CD68 cells into the lungs. Despite available in vitro and in vivo data suggesting that chemokine blockade may have a beneficial effect on airway hypersensitivity and asthma, to the best of our knowledge, there have been no studies that have found whether CCL1 blockade is involved in regulating the severity of pulmonary fibrosis in CGVHD models. Notably, we saw that CCL1 blockade with hMSCs resulted in a decrease in enhanced expression of CCL1, CCL17, and CCL22 induced by hMSCs in both the lungs and skin, indicating that CCL1 likely plays a critical role in the recruitment of immune effector cells into the lungs after administration of hMSCs. In particular, eosinophil trafficking and elevated IgE concentration in the lungs after hMSC treatment were also decreased by CCL1 blockade.

The current study did not evaluate the mechanistic basis by which the CCR8-CCL1 axis controlled and coordinated the paradoxical influx of inflammatory cells between the lungs and skin after hMSC injection. CCR8, the CCL1 receptor, is known to be critical for regulatory T (Treg) function. In mice, the chemokine receptor CCR8 is expressed principally on Treg cells and also notably on small fractions of Th2 cells, monocytic cells, and NK cells [42–44]. A similar expression pattern is seen in humans, in which CCR8 expression identifies CD4 memory T cells enriched for Foxp3^+^ regulatory and Th2 effector lymphocytes [45]. CCL1 may also have multiple effects on Treg and T effector biology. Therefore, blocking its activity may also have multiple end points. Moreover, while the combination of hMSCs with CCL1 blockade in an experimental CGVHD model provides preliminary data supporting further exploration, it is too early to see the effects of this work clinically translated.

## Conclusion

In this study, we showed that the administration of hMSCs in a xenogenic murine Scl-GVHD model effectively attenuated the severity of skin fibrosis, but paradoxically exacerbated pulmonary inflammation and fibrosis. CCL1 was identified to be the main contributing factor for MSC-induced recruitment of immune effector cells into the lungs. Concurrent treatment with hMSCs and a CCL1-blocking agent alleviated lung injury caused by hMSCs without detrimental effects on cutaneous CGVHD protection. We propose that the neutralization of CCL1 offers a potential means of preventing pulmonary complications induced by hMSCs when patients with Scl-GVHD or scleroderma are treated with hMSCs in the clinic.

## Supplementary information

**Additional file 1.** Supplementary methods and figure.

## Data Availability

The data that support the findings of this study are available from the corresponding author upon reasonable request.

## Abbreviations

BAL: Bronchoalveolar lavage
BM: Bone marrow
ECM: Extracellular matrix
ELISA: Enzyme-linked immunosorbent assay
EPX: Eosinophil peroxidase
GVHD: Graft-versus-host disease
HSCT: Hematopoietic stem cell transplantation
LN: Lymph node
MHC: Major histocompatibility complex
MMP: Matrix metalloproteinase
MSC: Mesenchymal stem cell
PTEN: Phosphatase and tensin homolog
SCL: Scleroderma
TGF-β: Transforming growth factor-beta
