# Supplementary material for: CCL1 blockade alleviates human mesenchymal stem cell (hMSC)-induced pulmonary fibrosis in a murine sclerodermatous graft-versus-host disease (Scl-GVHD) model
Source: Stem Cell Res Ther. 2020 Jun 26;11:254. doi: 10.1186/s13287-020-01768-7 (PMC7318460; doi:10.1186/s13287-020-01768-7)
Supplement: Supplementary file 1 — Additional file 1. Supplementary methods and figure. [file 13287_2020_1768_MOESM1_ESM.docx]

# **Supplementary materials and methods**

**Flow cytometric analysis of hMSC**

The phenotype of hBM- and hAD-MSCs were analyzed by flow cytometry using an LSRII (BD Pharmingen). The following mAb were used: phycoerythrin (PE)-conjugated anti-CD34, fluorescein isothiocyanate (FITC)-conjugated anti-CD45, allophycocyanin (APC)-conjugated anti-CD14, allophycocyanin-Cy7 (APC-Cy7)-conjugated CD11b, Peridinin Chlorophyll Protein Complex-Cy5.5 (PerCP-cy5.5)-conjugated HLA-DR, APC-conjugated anti-CD44, PE-conjugated anti-CD73, PE-conjugated anti-CD166 (BD Pharmingen).

**Differentiation of hMSC in culture**

Differentiation of hMSC into osteocytes, adipocytes and chondrocytes was done using ready-to-use differentiation media according to the manufactures instruction (R&D Systems, Minneapolis, MN). Osteogenic differentiation was verified using alizarin red staining, adipogeneic differentiation was verified using oil red o staining and chondrogenic differentiation was verified using alcian blue staining (sigma).

**Supplementary figure 1. Phenotype and differentiation potential of human BM- and AD-MSC.**

(A): Flow cytometry identification of cell phenotype. (B): MSCs were induced to differentiate toward osteogenic lineage and verified by alizarin red staining after induction, adipogenic lineage and verified by oil red o, and chondrogeneic lineage and verified by alician blue (magnification x 100).

**Supplementary figure 1.
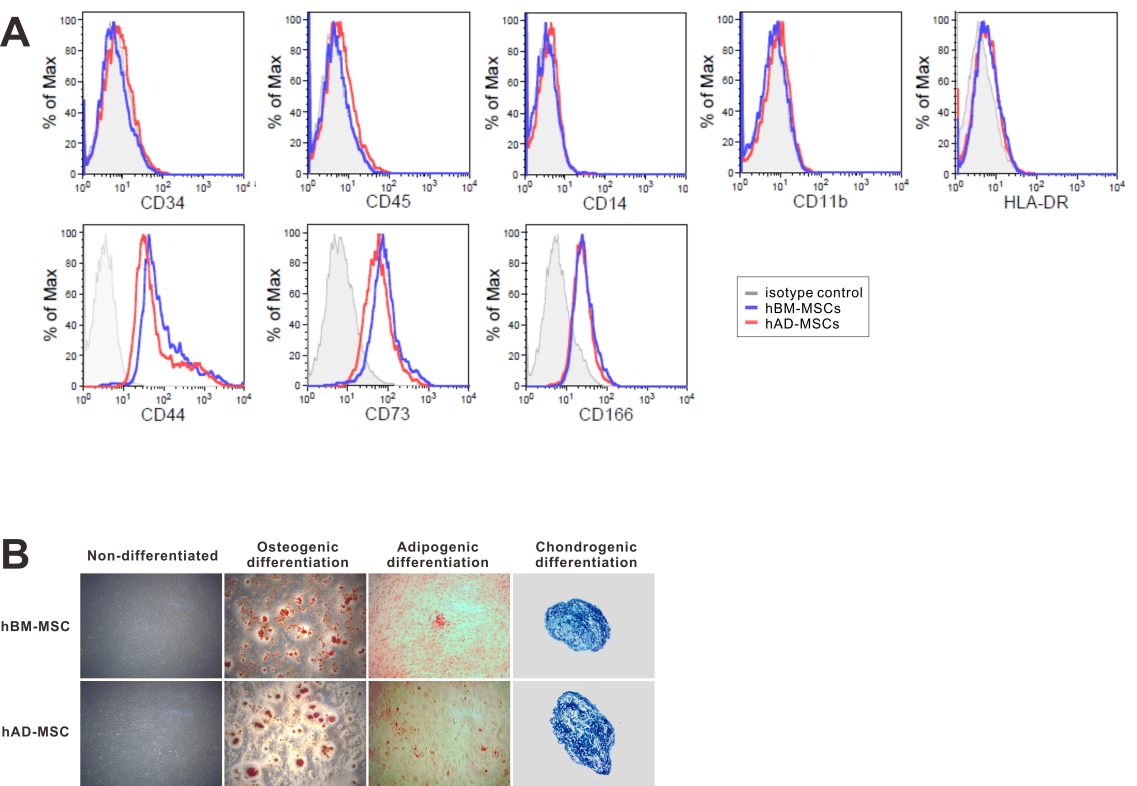
**
